# Immunosuppression in Glioblastoma: Current Understanding and Therapeutic Implications

**DOI:** 10.3389/fonc.2021.770561

**Published:** 2021-10-28

**Authors:** Benjamin T. Himes, Philipp A. Geiger, Katayoun Ayasoufi, Adip G. Bhargav, Desmond A. Brown, Ian F. Parney

**Affiliations:** ^1^ Department of Neurologic Surgery, Mayo Clinic, Rochester, MN, United States; ^2^ Department of Neurosurgery, University Hospital Innsbruck, Tirol, Austria; ^3^ Department of Immunology, Mayo Clinic, Rochester, MN, United States; ^4^ Department of Neurosurgery, University of Kansas, Kansas City, KS, United States; ^5^ Surgical Neurology Branch, National Institutes of Neurological Disorders and Stroke, National Institutes of Health, Bethesda, MD, United States

**Keywords:** immunosuppression, glioblastoma, myeloid - derived suppressor cell, extracellular vesicles, immunotherapy

## Abstract

Glioblastoma (GBM) is the most common primary brain tumor in adults an carries and carries a terrible prognosis. The current regiment of surgical resection, radiation, and chemotherapy has remained largely unchanged in recent years as new therapeutic approaches have struggled to demonstrate benefit. One of the most challenging hurdles to overcome in developing novel treatments is the profound immune suppression found in many GBM patients. This limits the utility of all manner of immunotherapeutic agents, which have revolutionized the treatment of a number of cancers in recent years, but have failed to show similar benefit in GBM therapy. Understanding the mechanisms of tumor-mediated immune suppression in GBM is critical to the development of effective novel therapies, and reversal of this effect may prove key to effective immunotherapy for GBM. In this review, we discuss the current understanding of tumor-mediated immune suppression in GBM in both the local tumor microenvironment and systemically. We also discuss the effects of current GBM therapy on the immune system. We specifically explore some of the downstream effectors of tumor-driven immune suppression, particularly myeloid-derived suppressor cells (MDSCs) and other immunosuppressive monocytes, and the manner by which GBM induces their formation, with particular attention to the role of GBM-derived extracellular vesicles (EVs). Lastly, we briefly review the current state of immunotherapy for GBM and discuss additional hurdles to overcome identification and implementation of effective therapeutic strategies.

## Introduction

Glioblastoma (GBM) is the most common primary tumor of the central nervous system (CNS) in adults, and carries with a dire prognosis, with median survival of just over 14 months in spite of maximal therapy including surgical resection, radiation, and chemotherapy with temozolomide (1, 2). This paradigm has remained essentially unchanged since 2005 and, while recent advances, including the addition of tumor treating fields (TTF) have shown some modest benefit, the overall course of the disease remains effectively unchanged (3). Effective new therapies are urgently required.

**Figure 1:**
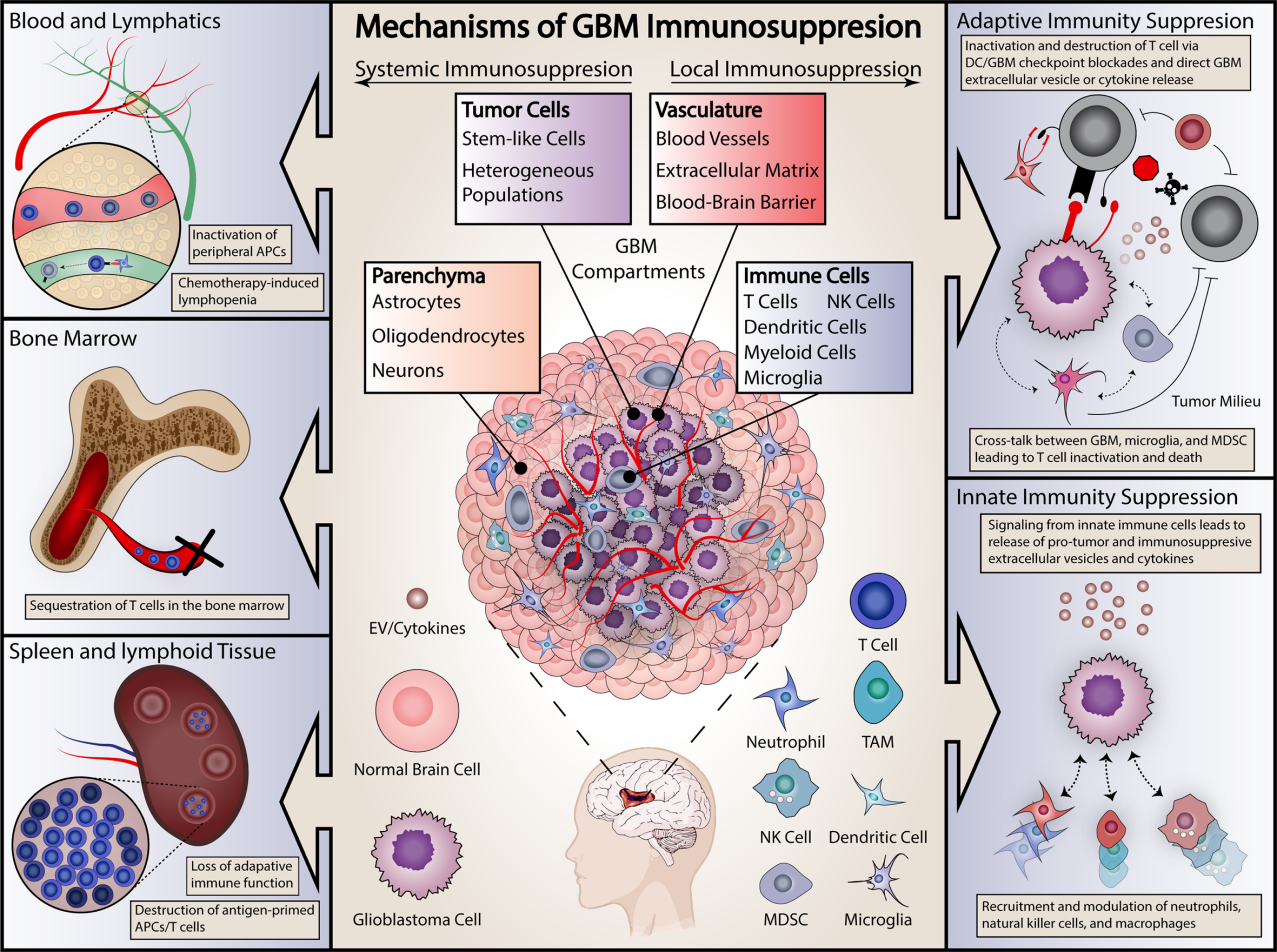
Summary of proposed mechanisms of GBM immunosuppression. Immunosuppressive effects are categorized as either systemic (on left) or local (right). Systemic effects are exerted on either the blood and lymphopoietic systems (including the bone marrow) or secondary lymphoid organs, including the spleen. Local effects include effects on both the adaptive and innate immune systems. Specific examples are included in each panel.

Immune-modulating therapies are promising for many diseases including cancer. These therapies range from immunotherapies like check point inhibitors where the “brakes” are taken off the immune system in order to induce immune activation, to active immunotherapies like vaccines against cancer antigens, and even the use of oncolytic viruses to simultaneously kill tumor cells and stimulate anti-tumor immune responses. As a whole, immunotherapy has shown tremendous promise in cancer treatment, initially with hematologic malignancies and more recently in solid tumors. This includes several cancer types such as melanoma that previously carried a devastating prognosis (4–6). These therapies hinge on activating and enhancing the immune system’s natural role in tumor surveillance and regulation, with specific treatments ranging from antibodies directed against specific tumor antigens, to tumor-derived vaccines, to chimeric-antigen receptor (CAR) T cells, to immune checkpoint inhibitors that seek to disinhibit the immune response against tumor cells (7–10). All of these promising strategies depend upon the underlying integrity of the patient’s immune system in order to be of benefit. Successful cancer immunotherapy is dependent on existence of an intact and functional immune system. However, GBM patients frequently exhibit profound local and systemic immunosuppression, limiting the likely efficacy of these therapeutic strategies (**Figure 1**) (11–14). This overt immunosuppression is a critical barrier to improving patient survival through immunotherapy. Without targeting this immunosuppression in GBM, most immunotherapies seem destined to fail. Indeed, several prominent clinical trials of immunotherapies in GBM have failed to demonstrate therapeutic benefit (15–19).

Understanding the mechanisms of immune dysfunction is essential to effectively employing immunotherapies in GBM, yet the nature of these mechanisms remains surprisingly elusive. Tumor-mediated immunosuppression in GBM is unique in that it is severe, multifaceted, and simultaneously affects the tumor-microenvironment and peripheral immune organs even though the tumor itself is limited to the central nervous system (**Figure 1**). In this review, we summarize current knowledge regarding mechanisms of immunosuppression in GBM and offer insights into future immunotherapeutic avenues for this devastating disease.

## Epidemiology and Current Treatment

GBM is the most common primary brain tumor, with an annual incidence of 3.19 per 100,000 patients diagnosed annually in the United States (20). Median age of onset is 64 and the disease has a predilection for Caucasian males (20). Current therapy entails maximal safe surgical resection followed by radiation (typically 60Gy over thirty fractions) with adjuvant temozolomide chemotherapy (2). With these maximal interventions, median survival remains just over 14 months with a 2 year survival of under 30% (21, 22). Additional treatments such as the addition of tumor-treating fields (TTF; locally delivered alternating electrical fields) to first line therapy or bevacizumab (anti-angiogenic therapy directed at vascular endothelial growth factor) in recurrent disease provide some modest benefit, but little has changed in the therapeutic paradigm in nearly fifteen years (3, 23). Certain molecular subgroups such as isocitrate dehydrogenase (IDH) mutant or O^6^-methylguanine-DNA methyltransferase (MGMT) promoter methylated tumors have been correlated with increased survival, but survival in even these cases remains poor long-term (24–26). Occurrence is sporadic, with few environmental or genetic risk factors identified in most cases.

## Immune Surveillance in Cancer and Glioblastoma

Fundamentally, cancer develops in part from a failure of normal immune surveillance. This has traditionally been described as proceeding through three phases: elimination, equilibrium, and escape (27). In the course of normal cell growth and tissue maintenance, cells suffer mutations from mitotic errors or environmental insults, predisposing them to neoplasia. In a healthy immune system, these cells are detected and deleted before tumor formation (the elimination phase) (27, 28). This proceeds through several mechanisms. Mutated cells can present neoantigens on the major histocompatibility complexes (MHC) on their cell surface, failing to register as presenting ‘self’ antigens by circulating natural killer (NK) or CD8 T cells, resulting in their targeted removal (29). Focal tissue disruption caused by tumor growth causes the release of inflammatory mediators and alarmins, triggering an innate immune response and tissue remodeling, which can create a hostile microenvironment for tumor growth (30).

Historically, the immune system’s role in tumor surveillance was considered controversial. Because autoreactive T and B cells are deleted during development to prevent autoimmunity, the idea of beneficial deletion of host cells by mature immune cells in the periphery was considered anathema to basic function of the immune system. However, accumulating evidence such as increased tumor development in immunodeficient animal models lent credence to the idea that the immune system serves as a check on tumorigenesis (27, 31). Similar results were found in immunosuppressed human patients (32). Seminal experiments demonstrating the major histocompatibility complex (MHC)-match dependence for transplantable tumors and the ability of vaccines against tumor antigens to protect from subsequent tumor inoculation further supported the crucial role of the adaptive immune system in anti-tumor immunity (33, 34). Understanding of the importance of this role ultimately led to the discovery that tumors utilize critical immune checkpoint molecules such as programmed death ligand 1 (PD-L1) and cytotoxic T lymphocyte associated protein 4 (CTLA-4) as a means to prevent immune activation towards cancer cells (35, 36). Such inhibitory molecules are one of the key mechanisms by which tumor cells can impede effective immune responses, and hence blocking this inhibition has become a pillar of modern cancer immunotherapy.

## Immunosuppression in the Tumor Microenvironment in GBM

Tumor growth leading to cancer fundamentally requires evasion of and escape from immune surveillance. GBM tumor cells sometimes downregulate MHC expression in order to avoid neoantigen presentation, though this may be more common in other cancer types such as melanoma (37, 38). Tumor cells themselves lose expression of MHC class I, which is expressed nearly ubiquitously by cells and is critical ‘self’ *versus* ‘non-self’ distinction by the immune system (39, 40). Loss of MHC class II, which is typically more selectively expressed by antigen presenting cells (APCs) and is essential for cross-presentation of antigens to adaptive immune cells, has also been described in GBM, particularly microglia, underscoring the broader immunosuppressive effects of the tumor (41, 42). The GBM microenvironment is rich in immunomodulatory factors, including transforming growth factor β (TGF-β), interleukin 10 (IL-10), and prostaglandin E-2 (PGE2) (43–46). Increasing evidence suggests that these immunosuppressive factors, particularly TGF-β derived from the GBM cells themselves, support transition of brain resident/infiltrating immune cells such as microglia and tumor infiltrating myeloid cells to an immunosuppressive phenotype that allows aggressive tumor growth and progression while blocking anti-tumor immune responses (47, 48). Immunomodulatory surface ligands including PD-L1 are also frequently expressed by tumor cells, including GBM, reducing anti-tumor immunity and promoting T cell exhaustion and anergy (49). Other immunomodulatory signals, including IDO and MIF, have also been reported in GBM (50–52). Additional GBM-derived factors such as interleukin 6 (IL-6) help recruit myeloid cells, prompt a shift in the immune response from inflammatory anti-tumor responses to anti-inflammatory and wound-healing type responses, reduce the ability of immune cells to effectively destroy tumor cells, and can lead to tissue remodeling to create a site of relative immune privilege and thereby preventing immunologic access to the tumor cells (14, 53, 54).

In GBM particularly, this is associated with a large amount of vascular remodeling and abnormal angiogenesis promoted by vascular endothelial growth factor (VEGF), which has extensively been investigated as a therapeutic target in GBM resulting in the regular use of the anti-VEGF antibody bevacizumab in GBM treatment, though this may have only modest impact on overall survival (if any impact at all) (23, 55–57). Hypoxia within the tumor microenvironment has also been implicated in impairing immune cell function, particularly through increased expression of hypoxia-inducible factor 1-α (HIF1-α), whose upregulation is associated expression of immunomodulatory proteins including PD-L1 in other cancers (58–60). In GBM, exposure to GBM cell conditioned-media in the presence of hypoxia has been shown to induce the formation of immunosuppressive myeloid-derived suppressive cells at a higher rate than normoxic conditions (61). With tumor cell division and a shift in the microenvironment, a stable nidus of tumor cells is able to persist in spite of immune surveillance (the equilibrium phase).

Finally, immunologic control ultimately breaks down as tumor cell proliferation overwhelms the ability of the immune response to remove cancerous cells, especially as this response is attenuated by the aforementioned factors. This final stage is termed ‘escape,’ and tumor growth proceeds relatively unchecked. In many cancers this manifests as distant metastasis formation in addition to continued growth at the primary tumor site. In GBM only local growth is typically seen, through leptomeningeal spread does occasionally occur (62). For GBM, unchecked disease manifests by uncontrolled tumor growth, which entails a persistent and expansive failure of the immune response to the tumor.

These local effects serve to suppress both the innate and adaptive components of the immune system. The GBM microenvironment, particularly through the release of IL-6 and the expression of PD-L1 and IDO-1, has been shown to induce the formation of regulatory T cells (Tregs) that blunt the anti-tumor T cell response (52, 63–66). Tregs release the immunosuppressive cytokine IL-10, inhibiting T cell proliferation and blocking anti-tumor immune responses, which further attenuates T cell cytotoxic activity and allows tumor growth. Recently, Miska and colleagues demonstrated that HIF-1α expression by Tregs was critical for their immunosuppressive functions within the GBM microenvironment (67). At the innate level, the microenvironment has similar effects on microglia and tumor-associated macrophages (TAMs), reducing their antigen-presenting capability and promoting a shift towards an immunosuppressive macrophage phenotype (68). A significant part to the tumor bulk in GBM has been identified as infiltrating neutrophils, and the have been proposed as an additional source of immune suppression through the expression of arginase 1 (69, 70). Finally, monocytic cells are associated with a pronounced immunosuppressive phenotype induced by the tumor, as discussed in further detail below.

## Systemic Immunosuppression in GBM

Broadly, the phenomenological evidence of tumor-mediated immune suppression can be divided into local and systemic effects. Despite the absence of systemic metastases, GBM patients frequently exhibit profound systemic immunosuppression (14). This is reflected in multiple ways, including reduced T cell counts and functionality. Indeed, CD4 T cell numbers in some GBM patients approach lows seen in patients with acquired immunodeficiency syndrome (AIDS) (12, 13, 71). In addition, GBM patients present with small secondary lymphoid organs compared to healthy volunteers (as measured by spleen volumes) and their blood-derived monocytes have lower class II MHC expression levels (12, 13). Smaller spleens, smaller thymi, reduced MHCII levels, and reduced CD4 T cell counts have been reproduced in both GL261 and CTIIA murine GBM models (11, 12). Moreover, sera isolated from glioma-bearing mice potently inhibits immune cells activation *in vitro* demonstrating presence of profound systemic immunosuppression in GBM (11). The thymus significantly involutes in glioma-bearing mice and bone marrow homeostasis is disrupted by accumulation of mature T cells within the niche (11, 12). Ayasoufi et al. demonstrated that serum isolated from glioma-bearing mice harbors a potent non-steroid factor that inhibits T cell proliferation *in vitro* (11). In short, GBM patients and glioma-bearing mice demonstrate a multifaceted systemic immunosuppression that affects both primary and secondary lymphoid organs.

The precise mechanisms underlying systemic immunosuppression in GBM are not well understood. It has been postulated that circulating tumor-derived cytokines could account for such overt immunosuppression. However, efforts quantifying circulating cytokines in GBM patients have failed to reveal levels sufficient to explain this profound systemic immunosuppression (13, 72). Others have suggested that systemic immunosuppression in GBM is simply a result of cytotoxic chemotherapy and other standard medications such corticosteroids used to treat cerebral edema. However, immunosuppression is seen in untreated GBM patients before receiving corticosteroids or chemotherapy (13). Additionally, untreated GBM-bearing mice exhibit the exact facets of immunosuppression observed in patients. While we do not know the exact mechanisms underlying systemic immunosuppression in this population, it remains a major barrier to effective immunotherapy in GBM patients. Simultaneously, this immunosuppression is a barrier to the success of any immune-modulating therapies introduced into this system. In order to get rid of the tumor, we must first reverse the immunosuppression.

## Effects of Standard Therapies on Local and Systemic Immunosuppression in GBM

Immunosuppression both systemically and locally can be increased by standard therapies for GBM. Temozolomide in particular is associated with myelosuppression which contributes to decreased lymphocyte counts (2). However, the effects of temozolomide on immune function are complex and several groups have suggested possible synergistic effects with immunotherapies, possibly through selective reductions in immunosuppressive regulatory T cells (73–75). Corticosteroids have immunosuppressive effects and are ubiquitous in the treatment of symptomatic cerebral edema in GBM patients. However, steroids are not the sole mechanism of immunosuppression as treatment naïve GBM patients also exhibit similar immunosuppression. Radiation therapy can potentially have negative in-field immunomodulatory effects, such as impaired wound healing post-surgery (76, 77). The effects of radiation therapy in the GBM microenvironment are also somewhat controversial. Radiation theoretically improves the accessibility of tumor neoantigens as tumor cells die, and in some cases may potentiate a systemic response to immunotherapy (78, 79). However, radiation also has multiple effects on immune cells in the tumor microenvironment. While some studies have suggested that radiotherapy increases T cell infiltration in GBM, Wang and colleagues recently noted an increase in M2 (anti-inflammatory-pro tumor growth) tumor-associated macrophages that correlates with relapse following radiation and likely contributes to an immunosuppressive microenvironment as well as resistance to radiation therapy (80–82). Radiation necrosis post-treatment, which involves formation of fibrotic tissue and vascular abnormalities, can also present an additional barrier for immune cells to traverse to effectively encounter residual tumor cells (83).

Circulating immunosuppressive cytokines are not sufficiently elevated in GBM patients to account for their systemic immunosuppression (13, 14, 72). This is particularly curious in GBM where, unlike many cancers, the primary tumor virtually never metastasizes. This suggests that systemic effects result either from previously unappreciated tumor-secreted and/or brain derived factors, or by the local induction of immunosuppressive cells that subsequently exert systemic effects.

## Immunosuppressive Monocytes Including Myeloid-Derived Suppressor Cells in GBM

While the precise mechanisms of tumor-mediated immune suppression remain an area of active investigation, many studies have pointed to the induction of immunosuppressive monocytes such as myeloid-derived suppressor cells (MDSCs) as a key immunosuppressive mechanism in GBM (13, 72, 84). MDSCs are a heterogenous population of monocytic cells that have been implicated in tumor-mediated immune suppression in multiple cancers including glioblastoma (85–88). These cells exert their effects locally through the release of immunomodulatory cytokines including IL-10, TGF-β, IDO-1, and arginase, curtailing the adaptive immune response (52, 72, 84–86). Precise definitions of MDSCs remain in flux, with most definitions combining surface marker profile and a functional measure of immune suppression such as inhibiting T cell activation/proliferation or release of immunosuppressive cytokines such as IL-10 (61, 72, 84). The current key MDSC categories include monocytic (mMDSCs) and granulocytic (gMDSCs). In addition, a number of other types of immunosuppressive monocytes including early MDSCs and non-classical monocytes have been described.

A growing literature has been concerned with mMDSCs in glioblastoma, which have a surface marker profile in humans characterized by CD14 expression combined with low HLA-DR expression (13). Loss of CD14 and CD15 expression is also frequently used to help differentiate mMDSCs from gMDSCs (89). Both mMDSCs and gMDSCs are derived from CD14+ monocytes. Increased populations of these cells have been described in the tumor microenvironment in a number of cancers, including breast, ovarian, and lung cancers (90–92). They have also been reported in glioblastoma, where Woichiechowsy and colleagues initially described reduced HLA-DR expression and cytokine release in monocytes collected from GBM patients (93, 94). These cells induce immune suppression by inhibiting conventional T cells, releasing immunosuppressive cytokines including IL-10 and TGF-B, and upregulating immunosuppressive PD-L1 and IDO-1. They have been found systemically as well as within the tumor microenvironment (72, 95). A similar population of cells is defined in mice by high levels of Ly6-C expression and absent Ly6-G expression in Gr-1+ myeloid cells (96).

Granulocytic MDSCs (also called polymorphonuclear MDSCs, or PMN-MDSCs) are frequently discussed, albeit less well-defined, in GBM. They typically lose CD14 expression while retaining high levels of HLA-DR and expressing CD15 and CD33 (86, 97). These cells have also been described in a number of cancers and induce functional immune suppression (98). Some studies have speculated that these cells are of neutrophilic rather than monocytic origin, however other evidence points more strongly to these cells also deriving from monocytes (99–101). Immunosuppressive neutrophils may be a separate, distinct entity or may overlap with gMDSCs but overall neutrophilia has long been described in multiple cancers, including GBM (69). Significant challenges in defining specific markers to effectively distinguish neutrophils from gMDSCs (both express CD15, which is commonly used to distinguish gMDSCs from mMDSCs) has led to significant ambiguity to the relative contributions of these cell types in immune suppression, with a recent study by Negorev and colleagues suggesting that common techniques used to isolate peripheral blood mononuclear cells (PBMCs) for the study of circulating MDSCs may be susceptible to high levels of neutrophil contamination (102). Another recent study has put forth LOX-1 as a potential gMDSC-specific marker (99). The murine analog of gMDSCs express low levels of Ly6-C and high levels of Ly6-G (96).

The relative importance of mMDSCs and gMDSCs in glioblastoma, and in cancer in general, is the subject of debate. The relative fractions of MDSCs induced seem to differ in human disease and murine models, with the latter having a strong predilection for gMDSC development, while the relative proportion in human disease has been more ambiguous (103, 104). This may be related to some evidence of sexual dimorphism in MDSC responses in GBM, as some immunocompetent murine models require the use of female mice (105). A recent study by McKelvey and colleagues also suggests a temporal evolution in MDSC populations infiltrating tumor, with an initial peak of gMDSC following tumor implantation and then an accumulation of mMDSCs (106). gMDSCs may make up the bulk of tumor-infiltrating MDSCs in GBM, while mMDSCs can be detected in the peripheral blood of GBM patients (89). The tumor microenvironment likely plays a significant role on a case or disease-specific basis, as the relative presence of granulocyte colony-stimulating factor (G-CSF) and granulocyte-macrophage colony-stimulating factor (GBM-CSF) influences the development of gMDSCs or mMDSCs, respectively (104, 107, 108). Distinguishing gMDSCs from tumor-infiltrating neutrophils remains an area of debate as well, and distinguishing MDSCs in general from tumor-associated macrophages or microglia remain an ongoing challenge.

While mMDSCs and gMDSCs have been traditionally discussed as predominant types of immunosuppressive cells in cancer, there is an increasing understanding that immunosuppressive monocytes as a group are likely far more heterogenous than these categories would imply. A number of recent studies have described early MDSCs (eMDSCs), which may represent a traditional state into a mature MDSC subtype (96, 109). A growing body of evidence, including work by our own group, has pointed toward programed death ligand 1 (PD-1) positive non-classical monocytes, (previously defined as CD14 mid-to-high, CD16+ cells) as an important mediator of tumor-derived immune suppression (84). A recent murine study by Strauss and colleagues demonstrated that selective deletion of PD-1 in myeloid cells, in contrast to T cells, lead to better tumor control in a melanoma model (110). Given the heterogeneity of these populations of immunosuppressive monocytes, it may prove difficult to precisely define the relative importance of each in systemic immune suppression, and indeed, this could vary from cancer to cancer and even from patient to patient depending on the precise biology of the tumor. There is significant overlap in the means by which different types of immunosuppressive monocytes exert their effects. Ultimately, an appreciation of the diversity of immunosuppressive monocytes is critical for developing effective therapeutic strategies, as an effective approach need to adequately address multiple potential sources of immune suppression, rather than focusing exclusively on a given MDSC subtype.

## Mechanisms of Tumor-Mediated Induction of Immunosuppressive Monocytes

**Figure 2:**
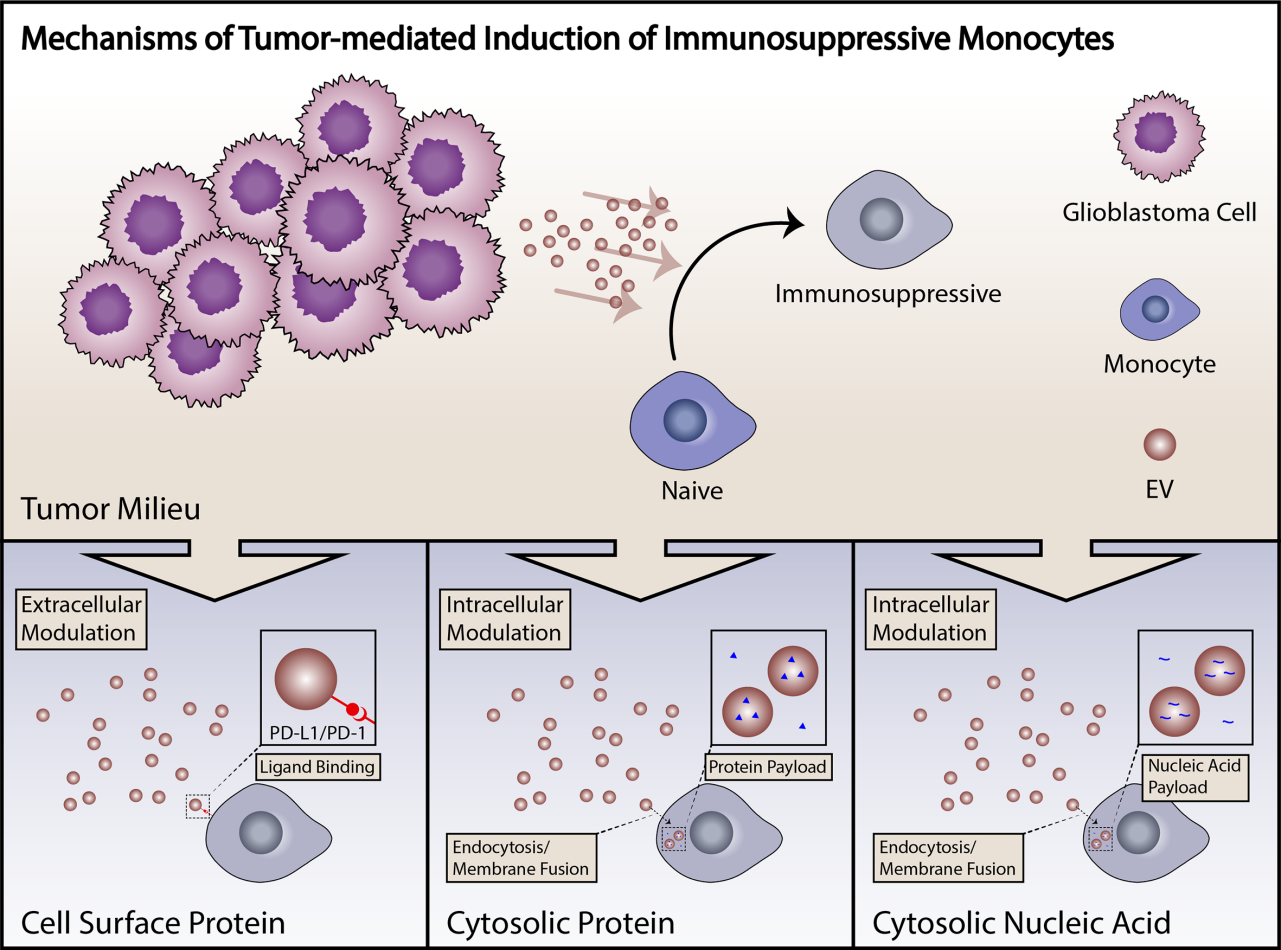
Summary of mechanisms of induction of immunosuppressive monocytes. Induction of immunosuppressive monocytes by GBM tumor cells can proceed through a number of different mechanisms, including direct cell surface-mediated signaling, uptake of proteins with subsequent cytosolic effects, or signaling by tumor-derived small RNAs. Tumor-derived EVs are capable of signaling by any of these mechanisms.

The tumor microenvironment in GBM is inherently immunosuppressive. GBM tumors release immunosuppressive cytokines including TGF-B, prostaglandin E_2_, and other immunosuppressive cytokines (14). PD-L1 expression is frequently elevated in tumors, preventing an effective anti-tumor immune response (49). This milieu causes a shift in the profile of resident immune cells towards a more permissive Type 2 response or in some cases a frankly immunosuppressive phenotype (111). This behavior applies to monocytes/macrophages, as detailed above, but has also been described in T cells (112). In many cases the exact mechanisms are not yet well understood (**Figure 2**).

**Figure 3:**
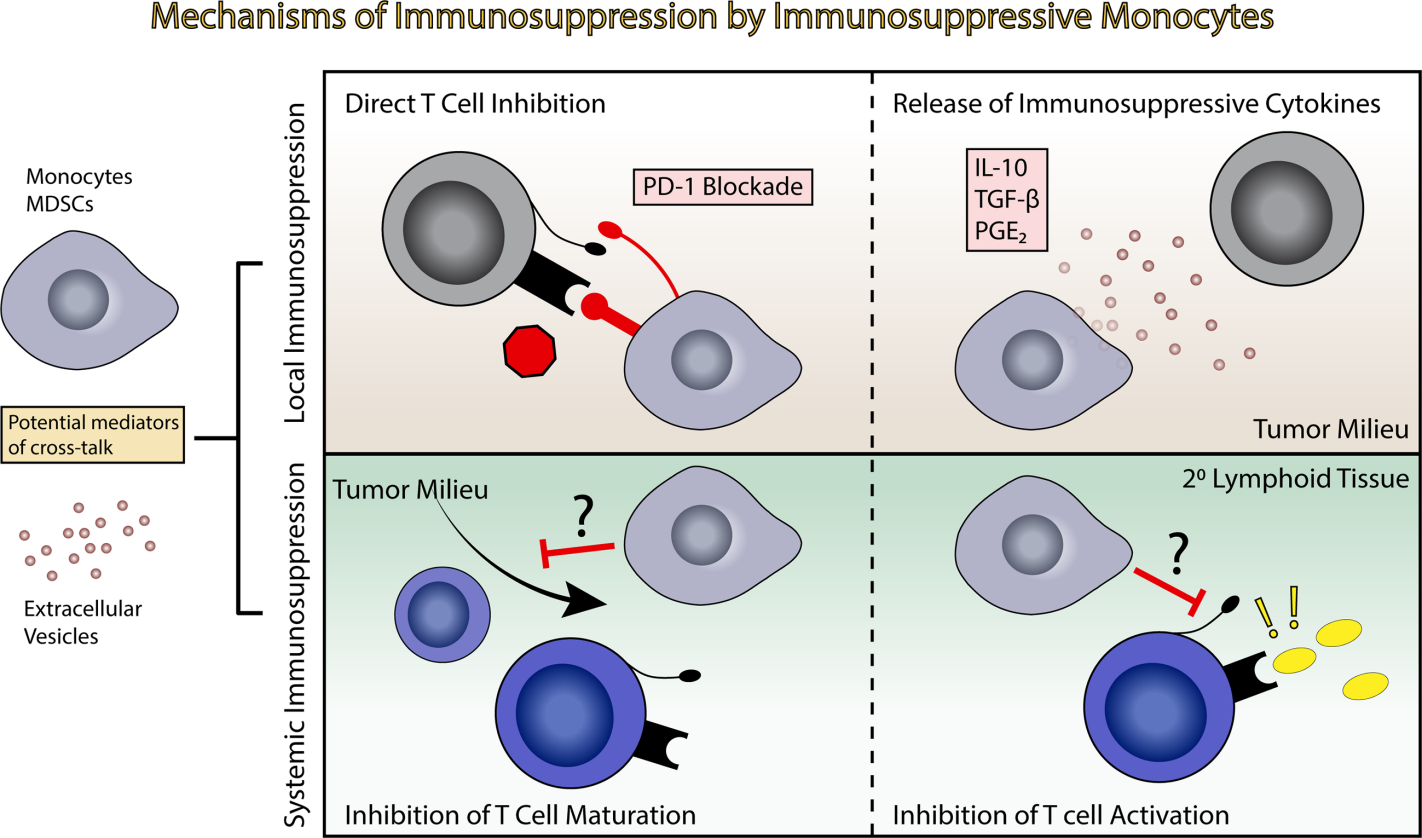
Induction of immunosuppression by immunosuppressive monocytes. Immunosuppressive monocytes and MDSCs potentially exert both local and systemic effects leading to immune suppression. This can include direct T cell inhibition and release of immunosuppressive cytokines in the tumor milieu (top panels), or inhibition of T cell maturation or inhibition in the primary and secondary lymphoid tissues (bottom panels).

Increasing evidence suggests that tumor-derived extracellular vesicles (EVs) are major mediators of tumor-induced immune suppression in GBM (84, 113). EVs are small lipid bilayer-encapsulated particles shed from the surface of all cells. These are released through several mechanisms, including direct membrane budding (microvesicles or large EVs, > 100 nm) and endocytototic/Golgi apparatus-derived exocytotic pathways (exosomes or small EVs, < 100nm). These particles are shed in large volumes by tumor cells, are present within the local microenvironment, and have the potential to enter systemic circulation. EVs are biologically active particles carrying both membrane-bound receptors and soluble proteins which can be functionally delivered to target cells, either through cell surface interactions, endocytotic uptake, or direct membrane fusion. EVs also carry coding mRNA and short non-coding RNAs including microRNAs, pi-RNAs and y-RNAs, that can carry out biological functions when delivered to target cells (**Figure 2**) (114). Our group has recently described the role of PD-L1 expression in GBM-derived EVs in the induction of PD-1+ non-classical monocytes, and demonstrated that EV-conditioning of healthy monocytes leads to the induction of an immunosuppressive phenotype (**Figure 2**) (84). Other groups have explored the role of GBM-derived EVs in direct inhibition of T cells (113). An increasing body of work from studies in other cancers points to EVs as a critical mechanism of tumor-derived immune suppression (115). All in all, EVs serve as an important immunosuppressive liaison between the tumor microenvironment and the peripheral immune system (**Figure 3**).

Immunosuppressive monocytes, similar to EVs, likely have the ability to exert immunosuppressive effects both locally and systemically, migrating from the tumor bed and entering systemic circulation, where they can influence T cell maturation and activation in the secondary lymphoid tissues (**Figure 3**) (116). MDSCs have been identified in the circulation of GBM patients, as well as in the bulk tumor.

## Immunotherapy in GBM

Current anti-tumor immunotherapies range from highly specific strategies to more general approaches. For example, antibodies directed against specific tumor fusion proteins or chimeric antigen receptor T cells (CAR T cells) provide specific and active immunity against specific cell types or tumor neoantigens, while checkpoint blockade inhibitors such as anti PD-1/PD-L1 or anti-CTLA4 increase the overall activity of the T cell response which consequently increases anti-tumor immunity (117). Additionally, vaccinations against tumor antigens and use of oncolytic viruses have also been put forward as immune-modulatory therapies for GBM.

GBM has particular features in addition to tumor-mediated immune suppression that present unique hurdles to effective immunotherapy. The mutational burden of GBM is middling on the spectrum of mutational burdens in cancer, meaning that it both lacks a defining mutation that presents a clear candidate for targeted therapy (the EGFRvIII mutation, which is frequently associated with GBM, is present in only 30% of tumors) and lacks the extensive genetic instability of high mutation burden tumors (e.g. melanoma) that present a range of immunogenic neoantigens and have shown a propensity for response to checkpoint blockade therapy (118, 119).

Drug (and immunotherapy) delivery also poses a challenge in GBM. The brain is no longer viewed as an immune privileged site. Microglia function as resident antigen presenting cells, T cells can traffic in and out of the brain, and recently-described lymphatic drainage allows for T cell surveillance of the central nervous system. However, the brain is certainly immunologically distinct site (120, 121). The blood-brain barrier (BBB) limits the penetration of both therapeutic agents and immune cells, making it difficult to deliver both drugs and cell-based therapies. Immune cell penetration into tumor most certainly occurs in GBM, but numbers of T cells seen infiltrating the tumor is relatively small. In parallel, neutrophils and myeloid-lineage cells make up the bulk of the tumor-associated immune cells (122–124). Direct delivery of therapeutic agents to the tumor through mechanisms including convection-enhanced delivery is one potential strategy for circumventing these anatomic challenges, and this technique could extend to the application of immunotherapies (125). Additionally, all immune-modulatory therapies rely on existence of an intact and functional innate and adaptive immune system. GBM patients are systemically immunosuppressed. These patients have very few T cells in circulations, small spleens, and their remaining T cells lack responsiveness against novel antigens. In fact, GBM patients do not respond strongly to flu vaccinations when compared to healthy controls demonstrating a challenge in vaccine design (126). Innate immune cells are also not optimally functional in these patients. The latter was demonstrated by lower levels of MHCII expression on blood-derived monocytes and the presence of suppressive MDSCs and neutrophils in circulations as discussed at length in the above section. In addition, serum isolated from mice with glioma was demonstrated by Ayasoufi et al. to potently inhibit proliferation of T cell *in vitro* (11). This further suggests that not only existing immune system in GBM patients is not functional, but also that putting healthy immune cells (i.e. CAR T cells) in the GBM patients’ circulation may render these cells not functional, as well. These together present even greater challenges to success of immunotherapies in GBM.

In spite of these challenges, a number of clinical trials have been undertaking exploring the efficacy of different immunotherapies for the treatment of GBM. These have been reviewed extensively elsewhere. However, in light of immunosuppression in GBM, it is perhaps not surprising that overall results from these studies have been disappointing (17, 127). Multiple studies involving checkpoint blockade inhibitors, most recently the CheckMate 143 study, which considered the use of the anti PD-1 antibody nivolumab *versus* bevacizumab for the treatment of recurrent GBM, failed to show a benefit (18). The only completed Phase III tumor vaccine study for GBM (ACT IV), which consisted of an EGFRvIII peptide in addition to treatment with GM-CSF and temozolomide, failed to show improvement in overall survival (17, 19). Earlier (Phase II) trials involving the use of oncolytic viral therapy, such as recombinant polio virus, have shown some promise, but more extensive trials are still required (128). These failed trials together with extensive accumulating evidence demonstrating multifaceted and systemic immunosuppression in GBM demonstrates that we must first reverse the immunosuppression before attempting to treat GBM patients with immune-modulating therapies. In the absence of such overt immunosuppression, endogenous anti-tumor-responses in combination with immunotherapies will likely produce successful results. Therefore, reversal of both local and systemic immunosuppression in GBM is the first step in designing a successful immunotherapy.

## Conclusion

Novel therapies for GBM remain urgently needed in order to improve prognosis for this uniformly fatal disease. Immunotherapy holds tremendous promise for revolutionizing cancer therapies, but results thus far in the treatment of GBM have been underwhelming. The reasons for this are multifactorial, ranging from the relative mutational burden of GBM to the unique physiology of the brain, but the significant immunosuppression seen in GBM patients undoubtedly plays a significant role. Indeed, it is impossible to rule out the potential efficacy of any trialed immunotherapies to date, as all have been tested in the context of patients with an abnormal immune system, setting them up for failure. Understanding and reversing this tumor-mediated immune suppression is critical to effective deployment of immunotherapies for GBM, whether it be checkpoint blockade or a tumor-derived vaccine. The mechanisms of this immune suppression remain an active area of investigation, but a growing body of evidence points to the induction of immunosuppressive immune cells, including MDSCs and non-classical monocytes, as essential mediators of immune suppression in GBM. Clearly understanding the induction of these cell types and therapeutically targeting their formation may be a critical avenue to treating GBM-mediated immune suppression. Following reversal of both local and systemic immunosuppression, endogenous anti-tumor responses and immunotherapies will undoubtedly produce favorable results.

## Author Contributions

BH contributed conception, design, and primary authorship of the manuscript. PG contributed writing, research, and revision support. KA and AB contributed writing, figure design, and revisions. DB contributed writing and revision support. IP contributed to conception and design as well as critical revisions. All authors approved the final version of the manuscript.

## Conflict of Interest

The authors declare that the research was conducted in the absence of any commercial or financial relationships that could be construed as a potential conflict of interest.

## Publisher’s Note

All claims expressed in this article are solely those of the authors and do not necessarily represent those of their affiliated organizations, or those of the publisher, the editors and the reviewers. Any product that may be evaluated in this article, or claim that may be made by its manufacturer, is not guaranteed or endorsed by the publisher.
